# Gait Adaptability and the Effect of Ocular Disorders on Visually Guided Walking in Parkinson’s Disease

**DOI:** 10.3233/JPD-230025

**Published:** 2024-04-23

**Authors:** Carlijn D.J.M. Borm, Debbie De Graaf, Bastiaan R. Bloem, Thomas Theelen, Carel Hoyng, Nienke de Vries, Vivian Weerdesteyn

**Affiliations:** a Department of Neurology, Hospital Group Twente ZGT, Almelo, The Netherlands; b Department of Neurology, Centre of Expertise for Parkinson and Movement Disorders, Radboud University Medical Centre, Donders Institute for Brain, Cognition and Behaviour, Nijmegen, The Netherlands; c Department of Ophthalmology, Radboud University Medical Centre, Nijmegen, The Netherlands; d Department of Rehabilitation, Donders Institute for Brain, Cognition and Behaviour, Radboud University Medical Centre, Nijmegen, The Netherlands

**Keywords:** Parkinson’s disease, gait adaptability, ocular disorders, non-motor symptoms

## Abstract

Gait disorders are a disabling feature of Parkinson’s disease (PD). To avoid falls, people with PD should be able to adequately adapt their gait. This requires correct response inhibition and integration of visual information. In this small pilot study, we investigated PD-related impairments in gait adaptability and the influence of ocular disorders thereon.

Compared with controls, persons with PD were less able to adapt their gait in unexpected situations (U = 21.5, *p* = 0.013), with only a small influence of ocular disorders on precision stepping (U = 6, *p* = 0.012 in the ML-direction and in the AP-direction, (U = 20, *p* = 0.456).

This shows that people with PD have more difficulty with precision stepping than healthy controls and experience more problems with adapting their gait. We found only a small impact of ocular disorders on successfully execute precision stepping. The ability to adapt gait, particularly in challenging environmental conditions or with impaired vision, may provide a useful assessment and training option for fall prevention in PD.

## INTRODUCTION

Gait disorders are among the most disabling symptoms in Parkinson’s disease (PD) because they significantly limit mobility and often result in falls and fall-related injuries [1]. To avoid falls when walking, people with PD not only need to generate a steady-state gait pattern, but they should also be able to flexibly adapt their gait to upcoming environmental changes. Adapting gait to changing situations requires response inhibition and integration of visual information [2, 3].

Previous studies demonstrated that people with PD approached and stepped over a fixed obstacle more slowly and with smaller steps than control participants, and also had a reduced capacity to avoid sudden obstacles [4, 5]. Yet, ambiguous findings were recently reported for foot placement accuracy when adapting gait to a pattern of stepping targets, possibly due to the targets also acting as visual cues, as a compensatory mechanism for underlying impairments [6].

In addition to gait disorders, co-existing visual and ocular disorders are highly prevalent in persons with PD [7]. Among these visual problems are diminished contrast vision, dry eyes, diplopia due to impaired ocular motor movements and cataract. One may expect these problems to further complicate gait adaptability, because of poorer retrieval of visual information from the environment. Indeed, blurred vision has been shown to affect obstacle crossing in older individuals, but the impact of ocular disorders on gait adaptability has not yet been studied in people with PD [8]. We expected this impact to be substantial, as people with PD rely more on visual information during the performance of motor tasks than healthy controls do [9]. Therefore, the aim of our small pilot study is to investigate the ability of persons with PD to adapt their steps to obstacles and regularly and irregularly-spaced visual targets, and to assess the impact of ocular disorders thereon. We hypothesized that: 1) persons with PD would show general performance decrements compared to healthy elderly, and 2) that persons with PD who manifest concurrent ocular disorders would perform worse on tasks that require precise step-to step adaptations of foot placement compared to persons with PD without ocular disorders.

## METHODS

This study is part of a larger study called ‘Visual Impairment in Parkinson’s Disease’ (VIP-study) [10]. The Medical Ethics Committee Arnhem-Nijmegen (NL58535.091.16) approved the study.

**Fig. 1:**
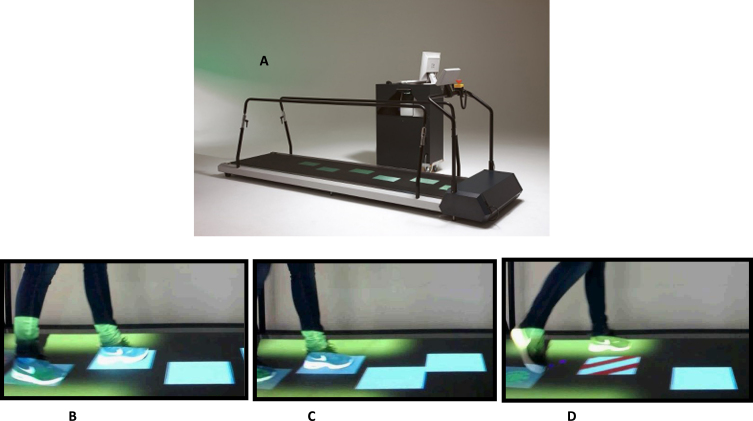
Study set up, motorized treadmill (C-mill). A) C-Mill, showing three tasks, each with a duration of 2.5 min at an individual’s comfortable walking speed (mean 3 km/h in the PD group). All tasks started with normal walking without stepping stones. B) Task 1; regular precision stepping, regular pattern of stepping stones on the belt. The participant placed the feet on the presented targets as exact as possible. C) Task 2; adaptive precision stepping. Visually guided stepping with irregular stepping stones. D) Task 3; bilateral obstacle avoidance. After 30 s, randomly and unpredictably, 20% of the stepping stones changed from white to a red/white striped rectangle, indicating an obstacle.

From this study population, 16 persons with PD (of whom 10 had ocular disorders) were recruited based on the highest and lowest scores on the visual impairment in PD questionnaire between May 2017 and December 2018. An extensive ophthalmological examination was performed specified in the study design of the larger study [10]. The relatively small number of patients with PD without any ocular disorders (*n* = 6) is explained by the high prevalence of patients with ocular disorders in our larger study [7]. Additionally, eight age-matched healthy controls were recruited from the partners of participants. The participants walked on a motorized treadmill (C-Mill) at their comfortable walking speed. In a baseline condition normal gait was established first. Three tasks where examined: 1) regular precision stepping: stepping stones (virtual tiles in the gait trajectory, upon which the foot had to be placed) were projected, in accordance with the normal gait pattern and foot size of each individual participant; 2) adaptive precision stepping: the stepping stones differed in pattern, with a maximum of 15% that corresponded to the normal gait pattern; and 3) obstacle avoidance: stepping stones were projected in a regular pattern, but now a steppingstone suddenly changed into an obstacle (see Fig. 1). Outcomes measures were the stepping precision in task 1 and 2 and the success rate of hitting the cues in task 3. The stepping precision is expressed as the variable error. This is quantified by the standard deviations of the anterior–posterior (AP) and medio–lateral (ML) distance between the center of the stepping stone and the corresponding center-of-pressure (COP) position at midstance, with lower values indicating a lower variable stepping error (i.e., higher stepping precision). We used the standard deviations of the distances between the COP position at mid-stance and the center of the stepping target (instead of the mean) as a measure of the accuracy of foot placement, because although the COP position at mid-stance is close to the center of the foot, it is not necessarily perfectly matched so there could be an error between the true center of the foot and the COP position at midstance. This depends on the way in which people walk.

### Statistical analysis

We compared PD patients and controls with respect to the variables; error of stepping precision, demographics, and the spatiotemporal parameters during the three gait tasks. The descriptive statistics are expressed in median and interquartile range, given the non-normal distribution of the data. Additionally, we tested if the success rates of effectively hitting the stepping stones and avoiding an obstacle was different between both groups. We used Chi-square-tests for categorical values and Mann-Whitney U tests for non-parametric continuous variables. Secondly, we looked at differences between PD patients with and without ocular disorders, for the same outcomes, using the same tests mentioned above. The Kruskal-Wallis test was used for comparison of the three groups. A *p*-value of *p* < 0.05 was considered as significant. Statistical analyses were conducted using SPSS 22.0 (SPSS Inc, IBM, Chicago, IL, USA) and SAS (SAS Institute Inc, Cary, NC) statistical software.

## RESULTS

**Fig. 2:**
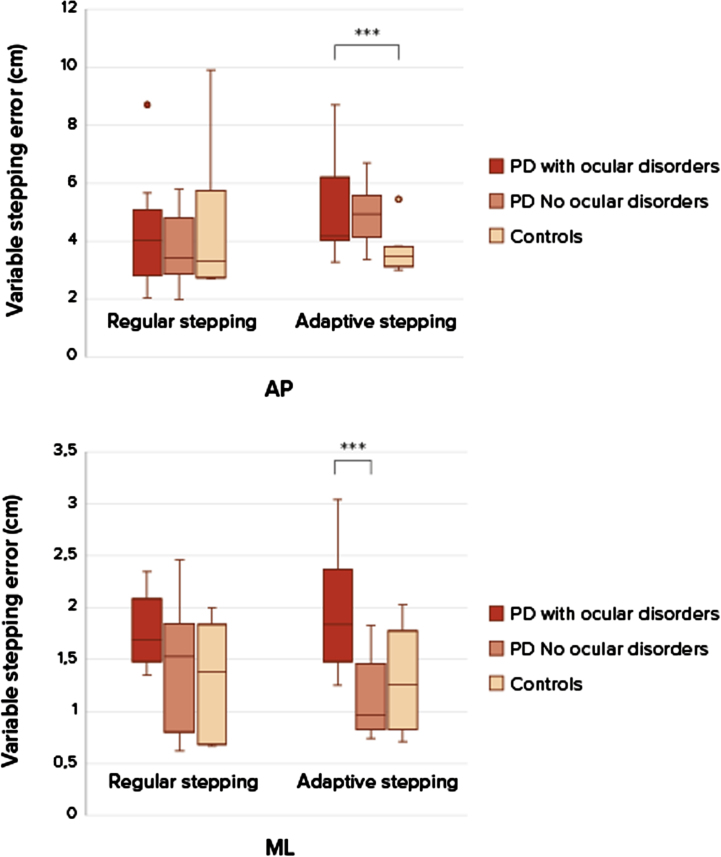
Boxplots of stepping accuracy between patients with ocular disorders, without ocular disorders and healthy controls. Differences in stepping accuracy (mean SD anterior-posterior (AP) and medio-lateral (ML) between regular stepping and an adaptive stepping compared between two groups patient with PD and healthy controls, at self-selected walking speeds. A *p*-value of *p* < 0.05 was considered as significant. Only significant differences are addressed with ***.***Significant difference between groups, PD compared to controls during adaptive stepping in the anterior-posterior direction. ***Significant difference between groups PD with ocular disorders and PD without ocular disorders in the medio-lateral direction.

**Fig. 3:**
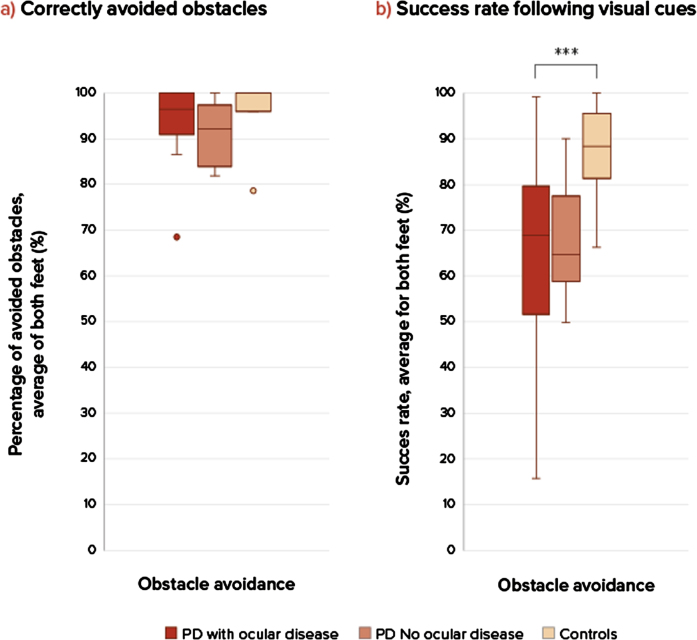
Success rates in obstacle avoidance. a) On the left side is the percentage of correctly avoided obstacles for each group, there were no significant differences. b) On the right sight is the success rate (percentage hit stepping stones during obstacle avoidance compared between three groups: PD patients with ocular disorders, without ocular disorders and healthy controls at comfortable walking speeds. A *p*-value of *p* < 0.05 was considered as significant. Only significant differences are addressed with ***. ***Significant difference between groups, PD compared to controls. Success rate during obstacle avoidance (U = 21, *p* = 0.011), PD compared to controls. There were no significant differences between the two PD groups and controls. NS, not significant.

The groups were comparable considering baseline characteristics (Table 1), although the group with ocular disorders showed a slightly longer disease duration and higher levodopa dose. The severity of ophthalmological diseases (based on the ophthalmological assessment) was based on a combination of literature and expert opinion and is depicted in the larger study article [7]. Only subjects with clinically relevant ocular disorders were grouped together in this sub study. Ocular disorders consisted mostly of diplopia, cataract, optic nerve degeneration, and dry eyes of moderate or severe severity. Al subjects had more than 1 ocular disorder. The stepping precision expressed as variable stepping error for the three groups shows no difference between the persons with PD and control group for task 1, regular precision stepping, in the AP-direction (U = 39.5, *p* = 0.668) and ML-direction (U = 28, *p* = 0.186) (Fig. 2). During the adaptive precision stepping, task 2, there is a significant difference. People with PD showed more difficulties in following the targets in AP-direction than controls (U = 21.5, *p* = 0.013). In the ML-direction this difference was not significant (U = 44, *p* = 0.302). Comparison among the PD groups showed that PD patients with ocular disorders had a decreased stepping accuracy compared to the group without ocular disorders (U = 6, *p* = 0.012 in the ML-direction but not in the AP-direction U = 20, *p* = 0.456). F Fig. 3 shows the success rate of avoiding obstacles and the ability to return to the normal gait pattern. Persons with PD had more problems returning to their normal gait pattern after avoiding an obstacle than in the control group, resulting in a lower hit rate of the stepping stones (U = 21, *p* = 0.012). There were no differences in success rates between the PD group with and without ocular disorders (U = 27, *p* = 1).

**Table 1:** Participants characteristics

	PD ocular disorders (*n* = 10)	PD no ocular disorders (*n* = 6)	Controls (*n* = 8)	Comparison within PD	Comparison controls vs. PD
				p*	p*
Age, median (IQR) [range], y	68 (6) [62–80]	68 (3) [65–68]	67 (9) [54–72]	1.00	0.697
Gender (male), n	70% (7)	83% (5)	50% (4)	0.395	0.605
Disease duration, median (IQR) [range], y	7 (9) [2–19]	3 (3.5) [2–7]	–	0.147
Hoehn &Yahr stage, median (IQR) [range]	2 (0) [2–3]	2 (0) [2–2]	–	0.647
Levodopa doses equivalent, median (IQR) [range], mg	719 (574) [90–1700]	350 (225) [300–600]	–	0.093
MoCA, median (IQR), [range]	27 (1) [24–29]	28.5 (3) [26–29]	–	0.560
GDS, mean (SD), [range]	6 (2) [4–9]	6.5 (1.6) [4–9]	–	0.544
Neurological assessment					
MDS-UPDRS part III ((/132), median (IQR) [range]	38 (15) [24–70]	38 (24) [29–61]	–	0.580
Falling (Yes), *n*	50% (5)	0% (0)	0% (0)	0.075
Visual function					
Total score VIPD-Q, median (IQR) [range]	21 (9) [3–30]	3.5 (4.8) [0–7]	6 (8) [0–10]	**0.003**	**0.012^**a**^**
Visual acuity OD, mean (SD) [range]	1.1 (.32) [0.63–1.6]	1.2 (.25) [1.00–1.60]	–	0.356
Visual acuity OS, mean (SD) [range]	1.1 (0.23) [0.8–1.6]	1.2 (0.13) [1.00–1.25]	–	0.356

PD, Parkinson’s disease; n., number of participants; IQR, interquartile range; SD, standard deviation; y, years; MoCA, Montreal Cognitive Assessment (score 0–30); GDS, Geriatric depression scale (score 0–30); PDQ-39, the Parkinson’s disease questionnaire-39 (score 0–100%); cm, centimeter; kg, kilogram; UPDRS MDS, unified Parkinson’s disease rating scale (total score 0–236), UPDRS part III (score 0–132); VFQ-25, visual function questionnaire-25 (score 0–100%); VIPD-Q, Visual impairment in Parkinson’s disease questionnaire (score 0–51); OD, oculus dextra; OS, oculus sinistra. For GDS, VIPD-Q, PDQ-39, and MDS-UPDRS, higher scores indicate worse functioning. For activities of daily living scale, VFQ-25, MoCA, lower scores indicate worse functioning. *p-value for the comparison of PD Ocular disease, PD no ocular disease and controls by analysis Mann-Whitney U or chi square tests for categorical variables. Fisher’s exact tests were abducted when rules applying to chi-square tests were not obtained. ^a^Indicates significant difference between PD with ocular disease and controls.

## DISCUSSION

In order to adapt gait properly to changing situations, response inhibition and integration of visual information is needed [3]. This small pilot study aimed to examine the gait adaptability of persons with PD and the influence of ocular disorders thereon. First, we hypothesized that persons with PD would show poorer precision stepping and lower success rates following visual cues during treadmill walking. Our study supports this hypothesis, as compared with controls, people with PD had more difficulty adapting their steps to irregular stepping stones, and when returning to a regular stepping stone pattern after avoiding an obstacle.

Our results confirm the notion that persons with PD have a reduced ability to adapt their gait in unexpected situations. Yet, in contrast to previous findings [4], the regular precision stepping task showed similar accuracy across all groups. This may be due to the combined cueing effects of the regular visual targets and treadmill walking, which was reported to normalize asymmetry and decrease gait variability [11]. Regular visual cues may alleviate the attentional burden of visual processing by focusing visual and attentional resources on task goals, which can improve gait characteristics (e.g., longer step length; and better gait initiation) [12, 13]. Real-world environments are visually complex and underlying attentional and visual function mechanisms are important for the visual cue response in PD [9]. Attention may also be required to compensate for motor or visual deficits that accompany PD. For example, increased saccade frequency with a visual cue may reflect compensation for visual deficits, as previous studies have reported an increased saccade frequency during visual search tasks in those with visual impairments [14]. However, in the adaptive precision stepping task, we found that persons with PD had difficulty adapting their gait to the visual cues, despite walking on the treadmill. This suggest that visual cues seem to function more as a visual ‘pacemaker’ than as a visual attractor for goal-directed foot placement. Of interest, poor precision stepping with a high stride-to-stride variability has been associated with disease progression and is indicative of increased fall risk in PD [15].

In addition, our results show that persons with PD have difficulties returning to a normal walking pattern after obstacle avoidance. This may be caused by their impaired ability to switch tasks. The obstacle avoidance task used in this study looks similar to a classical response inhibition task, from which we know that persons with PD exhibit impaired response inhibition [16]. However, avoiding an obstacle requires not just correct inhibition of a pre-planned step, but also replacement with a new one [3].

To our surprise, we found little evidence in support of the hypothesis that having ocular disorders would affect precision stepping and obstacle avoidance negatively. Our results only showed a difference in adaptive precision stepping in the medial-lateral direction between persons with PD with concurrent ocular disorders compared to those without ocular disorders. It may be speculated that the selective impact on ML stepping accuracy is related to persons with PD having particular difficulties controlling postural balance in this direction, as compared to the forward direction [17]. It could be argued that postural balance in the AP direction could have been corrected by lower limb muscles activation via the ankle strategy, while the ML component of postural sway is more complex and requires more energy [18, 19]. Any degradation in sensory information (e.g., vision) may therefore interfere more with ML than AP balance control, thus leaving less flexibility to make adjustments in ML foot placement for accurate stepping on the targets. The total impact of ocular disorders was smaller than expected, possibly because our cueing and obstacle tasks did not sufficiently mimic typical real-life scenarios, and therefore cannot be generalized to all variations and/or environments (e.g., outdoor versus indoor lighting, dim light, uneven terrain). Yet, our findings suggest that the ability to successfully execute precision stepping following visual cues is a rather robust gait mechanism, which is not heavily influenced by the various ocular disorders manifested by our study group.

We would like to address that the results of our pilot study should be interpretated with caution, since the small study groups could lead to bias with overreporting of statistically significant results and limited statistic options.

The ability to adapt gait, particularly in challenging environmental conditions or with impaired vision, may provide a useful assessment and training option for effective fall prevention in people with PD. Further research should address this issue in larger groups with a task representing daily situations and an increase in task complexity. Importantly, we do think that a trial resembling everyday situations is needed to investigate the role of visual problems and ocular disorders on gait and possible falls in persons with PD.

## Data Availability

The data supporting the findings of this study are available on request from the corresponding author.
